# CD36 aggravates ferroptosis in NK cells and dampens their anti-fibrotic activity in the liver

**DOI:** 10.3389/fimmu.2026.1825015

**Published:** 2026-05-14

**Authors:** Xiaokun Shen, Haitao Cao, Fujie Li, Wenya Wang, Xingshu Qi, Yarui Zhou, Bo Li, Qingjie Fan, Tao Yang, Shinan Li

**Affiliations:** 1 Liaoning Technology and Engineering Center for Tumor Immunology and Molecular Theranostics, Collaborative Innovation Center for Age-related Disease, Life Science Institute of Jinzhou Medical University, Jinzhou, Liaoning, China; 2 College of Basic Medical Science, Jinzhou Medical University, Jinzhou, Liaoning, China; 3 Department of General Surgery, The First Affiliated Hospital of Jinzhou Medical University, Jinzhou, Liaoning, China

**Keywords:** CD36, cell therapy, ferroptosis, liver fibrosis, NK

## Abstract

Liver fibrosis is a major global health challenge, and it is an independent risk factor for the development of hepatocellular carcinoma (HCC). Several methods have been explored to improve liver fibrosis progression, but effective preventive and therapeutic strategies remain limited. Natural killer (NK) cells can limit the activation of hepatic stellate cells (HSCs), but they have exhibited impaired antifibrotic properties in liver fibrosis, and the underlying mechanisms are not well understood. In this study, we found that CD36 was markedly upregulated in NK cells from mice with liver fibrosis. The CD36 expression was negatively correlated with activation markers of NK cells. The CD36^-^ NK cells exhibited elevated cytotoxic cytokine production and enhanced antifibrotic activity against HSCs. The adoptive transfer of CD36^-^ NK cells alleviated liver fibrosis progression. Mechanistically, CD36 induced reactive oxygen species (ROS) and lipid peroxidation. These consequently triggered ferroptosis in NK cells. The blockage of CD36 or inhibition of ferroptosis in NK cells effectively restored their antifibrotic properties against HSCs. In summary, our findings demonstrated that the antifibrotic properties of NK cells were regulated by CD36. These results provide evidence that targeting CD36 could restore NK cell function for liver fibrosis immunotherapy.

## Introduction

Liver fibrosis is a universal pathological feature of most hepatic pathogenetic processes and end-stage liver diseases. It is triggered by chronic insults that impair hepatocyte regeneration, activate inflammatory pathways, and recruit various cells (e.g., immune cells and hepatic stellate cells). These activated cells drive excessive extracellular matrix and collagen deposition. This results in a progressive, clinically silent loss of hepatocyte function. Uncontrolled fibrosis advances to cirrhosis and possibly to a tumor. Fibrosis is a major global cause of morbidity and mortality. Liver transplantation is the only effective clinical treatment, but it is severely limited by donor shortages (1). Cell therapy has emerged as a potential alternative, but multiple challenges hinder its clinical application (2, 3).

Driven by the continuous advancement of biotechnology and the in-depth elucidation of multiple molecular mechanisms and cellular crosstalk within the hepatic microenvironment, innovative cell therapy approaches have been actively developed for end-stage liver disease, a clinical outcome arising from decompensated liver fibrosis or cirrhosis. To date, many cell types (e.g., bone marrow mononuclear cells, endothelial progenitor cells, bone marrow mesenchymal stem cells, and macrophages) have been reported to have positive effects on the suppression and apoptosis of HSCs, collagen reduction, liver enzyme normalization, and proinflammatory cytokine decreases (3, 4). These results have indicated their remarkable therapeutic effects that have drawn attention.

NK cells exert dual regulatory effects on liver fibrogenesis by eliminating activated HSCs to attenuate fibrosis progression (5). However, excessive NK cell activation can also exacerbate liver tissue injury and promote fibrogenesis via pro-inflammatory cytokine release (6). It was suggested early in the research that HSCs in advanced fibrosis could inhibit NK cell function and cytotoxicity through the production of transforming growth factor-β (TGF-β) and the elevated expression of a suppressor of cytokine signaling 1 (SOCS1) (6). Therefore, NK cell-based therapy requires an evaluation of its efficacy as a potential therapeutic strategy for liver fibrosis.

A growing body of evidence has indicated that lipid metabolic reprogramming in both epithelial cells and nonparenchymal cells plays a pivotal role throughout the entire progression of fibrosis/cirrhosis-regeneration-HCC, thereby promoting the advancement of the disease (7). Therefore, NK cell-based therapy requires an evaluation of its efficacy as a potential therapeutic strategy for liver fibrosis (8). However, how CD36 influences NK cells has not been well explained. The aim of this study is to investigate whether CD36 affects NK cell function, as well as how it influences NK cell-based cell therapy strategies for liver fibrosis.

We concluded in this study that CD36 was highly expressed on NK cells of liver fibrosis mice. CD36-mediated ferroptosis dampened the NK cell effector function and impaired their anti-fibrotic ability. The adaptive transfer of CD36^-^ NK cells could improve liver fibrosis progression. In summary, our results provide novel theoretical support for the targeting of NK cell therapy to treat liver fibrosis.

## Materials and methods

### Animal model

6-8-week-old male mice were used in all the experiments. WT mice (C57BL/6J) were purchased from Beijing Vital River Laboratory Animal Technology Co. Mice were housed under specific pathogen-free conditions with a 12 h light/dark cycle and free access to food and water. Animal experiments and procedures were approved by the Animal Care and Use Committee of Jinzhou Medical University. Liver fibrosis was induced by i.p. injection of 2.5 ml/kg of 20% CCl_4_ (Macklin) dissolved in olive oil (aladdin), twice a week for totally 4 weeks. The mice were harvested 48h after the last injection of CCl_4_.

### Flow cytometry

The following antibodies were used for flow cytometry in this study. FITC-anti-NK1.1, -Granzyme B, -IFN-γ, -TNF-α; PE-anti-CD36, -NKG2D, -NKG2A, -IFN-γ, -Perforin; APC-anti- CD3, -CD36, -TIGIT; APC-CY7-anti-CD3, -CD69; BV421-anti-NK1.1; Zombie Fixable Viability Kit were purchased from Biolegend. Mononuclear cells of liver and spleen were stained with antibodies as indicated for surface antigens and intracellular cytokines staining as previously described (9). In some experiments, 1×10^6^ liver mononuclear cells were incubated with or without 10μg/mL α-CD36 (188150-1ea, Cayman) or 10μM Ferrostatin-1 for 18 h in 96-well plate for intracellular cytokines assay. The cells were collected, stained and detected on flow cytometer (BD FACSCelesta™) and analyzed using FlowJo software (Tree Star, Inc., Ashland, OR).

### ROS measurement

ROS of liver NK cells was detected by Reactive Oxygen Species Assay Kit (Beyotime Biotechnology). The data were collected on flow cytometer (BD FACSCelesta™) and analyzed using FlowJo software (Tree Star, Inc., Ashland, OR).

### Lipid peroxidation

3×10^4^ NK cells from Oil or liver fibrosis group were incubated with or without 10μg/mL α-CD36 (188150-1ea, Cayman) for 18 h in 96-well plate. Then, cells were incubated in a humidified chamber at 37°C with 5% CO_2_ for 30 minutes with lipid peroxidation sensor according to the manufacturer’s protocol (Abcam). After incubation, cells were washed and examined by flow cytometry. A decreased PE/FITC ratio is indicative of elevated lipid peroxidation levels.

### Immunofluorescence

Liver NK cells were purified by MACS (Miltenyi Biotec) and cultured with or without 10μg/mL α-CD36 (188150-1ea, Cayman) for 18 h as indicated. Cytocentrifuge preparations (3×10^4^ NK cells) were then made with Shandon Cytospin 4 (Thermo Fisher). Cells were fixed with 4% paraformaldehyde and washed with PBS. After permeabilization with PBS containing 0.25% Triton X-100 for 10 min, samples were incubated with 5% BSA for 30 min to block nonspecific antibody binding and then incubated overnight at 4 °C with primary antibodies against GPX4 (1:500; Affinity, DF6701) or COX2 (1:500; Affinity, AF7003), followed by washes with PBS and incubation with coraLite488-conjugated Goat anti-Rabbit lgG (H+L) (1:500, proteintech) for 1h at room temperature, then examined using an Leica ICS SP5 II.

Spleen NK cells were isolated using MACS (Miltenyi Biotec) and resuspended in 1× PBS at a final density of 4 × 10^4^ cells per 150 μL. As mentioned above, briefly, cells were incubated overnight at 4 °C with primary antibody against CD36 (1:50; Novus Biologicals, NB400-144). After three additional 1× PBS washes, slides were incubated for 1 h at room temperature with goat anti-rabbit IgG (H+L) highly cross-adsorbed secondary antibody conjugated to Alexa Fluor™ 546 (1:1000 dilution, Invitrogen), protected from light. Immunofluorescence imaging was acquired Leica ICS SP5 II.

### Cytotoxicity assay

The lactate dehydrogenase release assay was used to determine the cytotoxicity of NK cells against HSCs. Briefly, HSCs were seeded in 96-well plates (5×10^3^ cells per well) for 24 h, then culture with serum-free RPMI 1640 for 6 h to activate HSCs. Meanwhile, 5×10^4^ NK cells from oil or liver fibrosis were pretreated with 10μg/mL α-CD36 (Cayman) or 10μM Ferrostatin-1 (MCE) for 6 h in complete 1640 medium. The treated NK cells were cocultured with HSCs for another 12 h. The killing efficiency was measured using the lactate dehydrogenase Cytotoxicity Assay Kit (C0017; Beyotime) according to the manufacturer’s protocol. Cytotoxicity was calculated as follows: cell death ratio (%) = [(Asample-Acontrol)/(Amax-Acontrol)] ×100, where A is absorbance value.

### Adoptive transfer of NK cells

CD36^+^ NK cells or CD36^-^ NK cells from liver of fibrosis mice were isolated using flow cytometry (BD FACSAria™ III). Isolated NK cells (1×10^5^) were injected through the tail vein into fibrosis mice after the third time injection of CCl_4_, once a week for three times during CCl_4_ challenge.

### Histological analysis

The livers were removed and fixed in 10% formaldehyde before embedding in paraffin. Sections cut at a thickness of 5 μm were stained with H&E for histological analysis. The stage of liver fibrosis was scored according to the Ishak system. Sirius red staining was performed according to standard procedures. ImageJ software quantified sirius red-stained positive areas by analyzing characteristics such as collagen aggregation area, shape, and color.

### Quantitative PCR

Total RNA was isolated from liver tissue or purified NK cells and real-time qPCR was performed. The sequences of primers were listed as follows:

*Gpx4* (forward): 5′-TGTGCATCCCGCGATGATT -3′
*Gpx4* (reverse): 5′-CCCTGTACTTATCCAGGCAGA-3′
*Acsl4* (forward): 5′-CTCACCATTATATTGCTGCCTGT-3′
*Acsl4* (reverse): 5′-TCTCTTTGCCATAGCGTTTTTCT-3′
*Cox2* (forward): 5′- TGCACTATGGTTACAAAAGCTGG-3′
*Cox2* (reverse): 5′-TCAGGAAGCTCCTTATTTCCCTT-3′
*Col1a1* (forward): 5′-GAAACCCGAGGTATGCTTGA-3′
*Col1a1* (reverse): 5′- GACCAGGAGGACCAGGAAGT-3′
*Acta2* (forward): 5′-ACTACTGCCGAGCGTGAGAT-3′
*Acta2* (reverse): 5′-AAGGTAGACAGCGAAGCCAG-3′
*Des* (forward): 5′-GTTTCAGACTTGACTCAGGCAG-3′
*Des* (reverse): 5′-TCTCGCAGGTGTAGGACTGG-3′

### RNA sequencing

Total RNA (400ng) was extracted from CD36^+^ NK cells or CD36^-^ NK cells sorted from liver of fibrosis mice, followed by library preparation according to Illumina standard instruction (VAHTS Universal V6 RNA-seq Library Prep Kit for Illumina^®^). Agilent 4200 bioanalyzer was employed to evaluate the concentration and size distribution of cDNA library before sequencing with an Illumina novaseq6000. The protocol of high-throughput sequencing was fully according to the manufacturer’s instructions (Illumina). The raw reads were filtered by Seqtk before mapping to genome using Hisat2 (version:2.0.4). The fragments of genes were counted using stringtie (v1.3.3b) followed by TMM (trimmed mean of M values) normalization. Significant differential expressed genes (DEGs) were identified as those with a False Discovery Rate (FDR) value above the threshold (Q< 0.05) and fold-change >2 using edgeR software.

### Statistical analysis

All statistical analyses were performed using an unpaired Student’s *t*-test and One-way ANOVA analysis. The data are expressed as the mean ± SEM, and differences were considered statistically significant when *p* < 0.05 (*p < 0.05, **p < 0.01, ***p < 0.001, ****, *p* < 0.0001).

## Results

### CD36 is involved in the enrichment of lipid metabolism pathways in liver fibrosis

**Figure 1:**
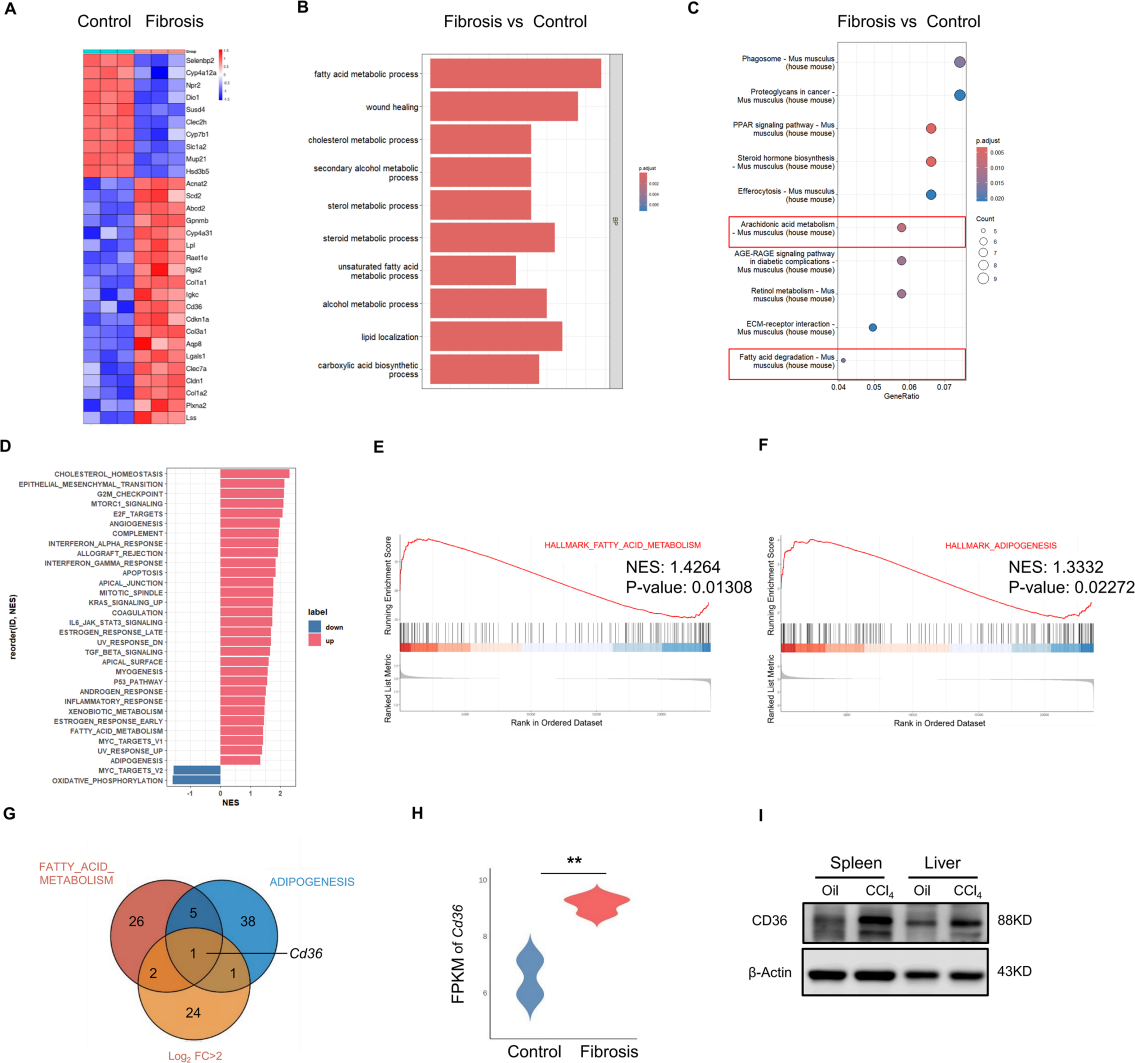
CD36 is increased in fibrotic liver and is enriched in lipid metabolism pathways. **(A)** A public dataset (GSE222576) was analyzed. Raw data were normalized using robust multiarray averaging (RMA, R package), and differences in gene expression were analyzed by screening for the genes with a Log_2_FC > 2 between the liver fibrosis group and the control group. **(B)** Gene Ontology (GO) term enrichment of the differentially expressed genes was analyzed using the Database for Annotation, Visualization, and Integrated Discovery (DAVID). The GO terms of the top 10 are shown. **(C)** The top 10 most enriched KEGG pathways associated with DEGs. **(D)** GSEA of the liver tissue from fibrotic vs control mice. The fatty acid metabolism pathway **(E)** and adipogenesis pathway **(F)** are shown. **(G)** The fatty acid metabolism pathway-related genes, adipogenesis pathway-related genes, and DEGs (log_2_FC > 2) were crossed. **(H)** The fragments per kilobase of transcript per million mapped reads (FPKM) of CD36 in the indicated livers. **(I)** Mononuclear cells of the livers and spleens from the control and CCl_4_-treated mice were subjected to western blot analyses to determine CD36 protein levels.

Previous research has indicated that alterations in hepatic lipid metabolism promote HSC activation and subsequent liver fibrosis by modulating the metabolic microenvironment. This includes the modulation of cellular energy metabolism and extracellular matrix synthesis (10). Based on this, we proceeded to investigate the role of lipid metabolism in liver fibrosis and its association with NK cell function. Initially, a public dataset (GSE222576) analysis revealed 28 genes (Log_2_FC > 2) that were significantly upregulated in liver tissues from fibrotic mice compared to controls (**Figure 1A**). Additionally, some genes (e.g., *Acnat2*, *Scd2*, *Lpl*, and *Cd36*) were associated with lipid catabolism. The Gene Ontology (GO) and Kyoto Encyclopedia of Genes and Genomes (KEGG) enrichment analyses identified significant enrichment of pathways related to lipid uptake and catabolism in the fibrotic group (**Figures 1B, C**). We then examined the fatty acid metabolism pathway, the adipogenesis pathway using a gene set enrichment analysis (GSEA) (**Figures 1D–F**), and differentially expressed genes (DEGs) (Log_2_FC > 2). We found that *Cd36* was significantly increased in the livers of fibrotic mice (**Figures 1G, H**). Furthermore, we isolated mononuclear cells from the liver and spleens of the control (Oil) and the CCl_4_-treated mice. The results showed that CD36 was significantly upregulated in the mononuclear cells after the CCl_4_ treatment. This result suggested that CD36 might participate in an impairment of immune surveillance in liver fibrosis (**Figure 1I**).

### NK cell dysfunction correlated negatively with CD36 expression

**Figure 2:**
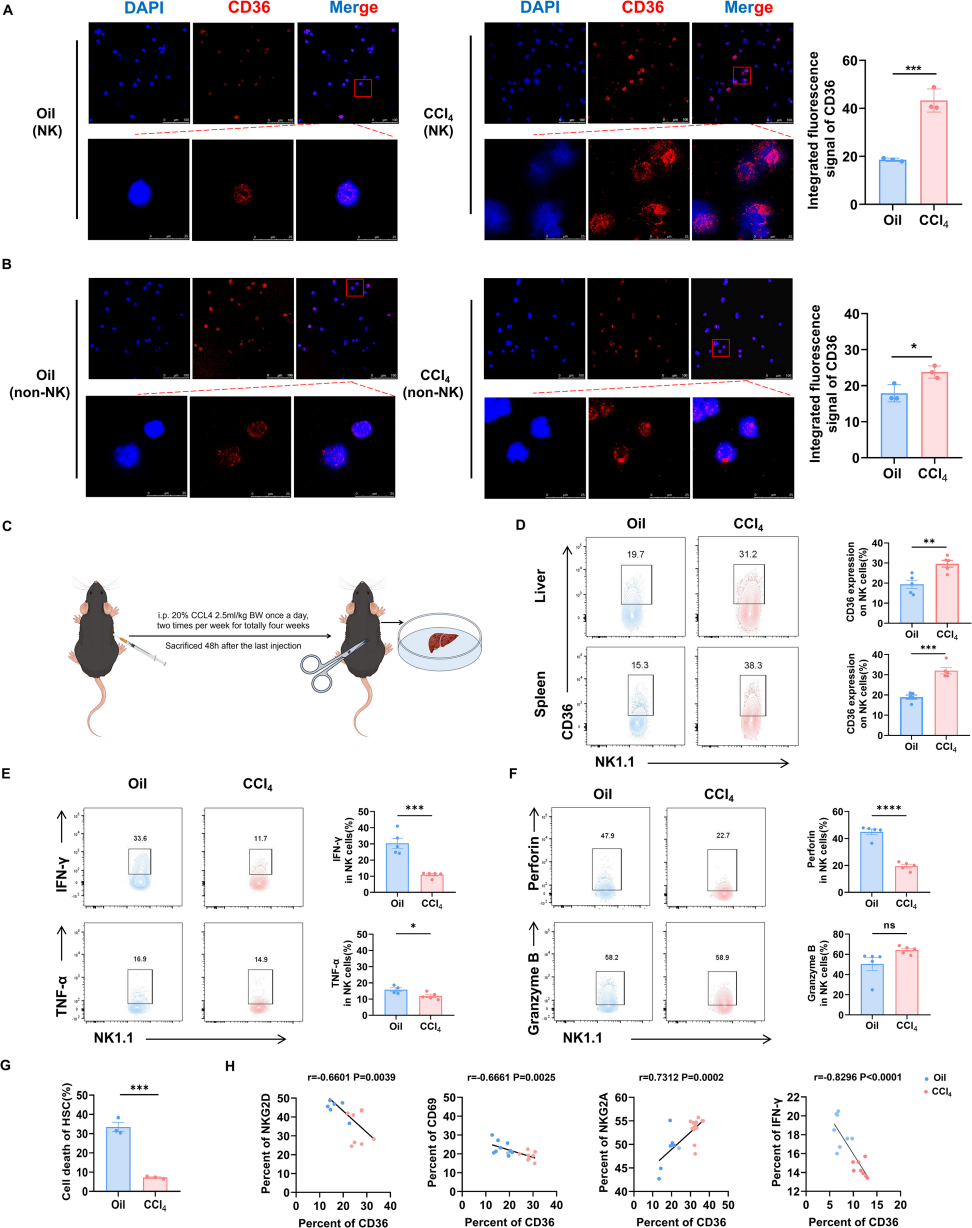
CD36 expression decreases cytotoxic cytokine production of NK cells. **(A)** Immunofluorescence images of CD36 expression on purified spleen NK cells with the indicated treatment. **(B)** Immunofluorescence images of CD36 expression on spleen mononuclear cells (excluded NK cells) with the indicated treatment. **(C)** Schematic of mouse treatment with CCl_4_. **(D)** CD36 expression of NK cells from the livers and spleens after the CCl_4_ treatment. **(E)** NK cells were analyzed for the expression of IFN-γ or TNF-α in the wild type (WT) and CCl_4_-treated mice. **(F)** NK cells were analyzed for the expression of perforin or granzyme B in WT and CCl_4_-treated mice. **(G)** Cytotoxicity of NK cells from the WT and fibrotic livers against activated mouse HSCs (NK cells: HSCs = 10:1). **(H)** CD36 expression of hepatic NK cells was analyzed using flow cytometry and plotted against that of NKG2D, CD69, NKG2A, and IFN-γ. Simple correlations were summarized using the Pearson’s correlation coefficients. Data are presented as means ± standard error of the mean (SEM). **, *p* < 0.01; ***, *p* < 0.001; ****, *p* < 0.0001.

Some studies had reported the effect of CD36 on CD8^+^ T cells in tumor, but how CD36 influenced NK cells remained not fully elucidated. Based on the above findings, we first sought to investigate CD36 expression on NK cells in liver fibrosis mice (**Supplementary Figures 1A–G**). Interestingly, spleen NK cells from the CCl_4_-treated mice expressed higher levels of CD36 compared with those from the control mice (**Figure 2A**). Additionally, mononuclear cells (non-NK) showed slightly increased CD36 expression (**Figure 2B**). We further confirmed these results using flow cytometry. The data showed that both liver and spleen NK cells from the fibrotic mice exhibited significantly higher CD36 expressions compared to those of the control mice (**Supplementary Figures 2A, B****;** **Figures 2C, D**). Furthermore, we also examined the expression of CD36 on other immune cell subsets and found that NK cells, particularly liver-resident NK cells (LrNK cells) (11), exhibited higher levels of CD36 (**Supplementary Figures 3A–F**). Previous studies have proved that NK cells from liver fibrotic mice exhibit a dysfunctional state (12), and this was confirmed by our results that showed that these NK cells secreted less IFN-γ, TNF-α, and perforin compared with those from control mice (**Figures 2E, F**). Moreover, the cytotoxic activity of NK cells from the liver fibrotic mice against activated primary mouse HSCs was decreased (**Figure 2G**). We further analyzed the correlation between CD36 and the activation markers of NK cells. CD36 was significantly negatively correlated with the expression of NKG2D, CD69, and IFN-γ, but it was positively correlated with NKG2A (**Figure 2H**). These results suggested that the impaired NK cell activity during fibrosis may be strongly associated with CD36 upregulation.

### CD36 blockage attenuated lipid peroxidation activation and rescued NK function

**Figure 3:**
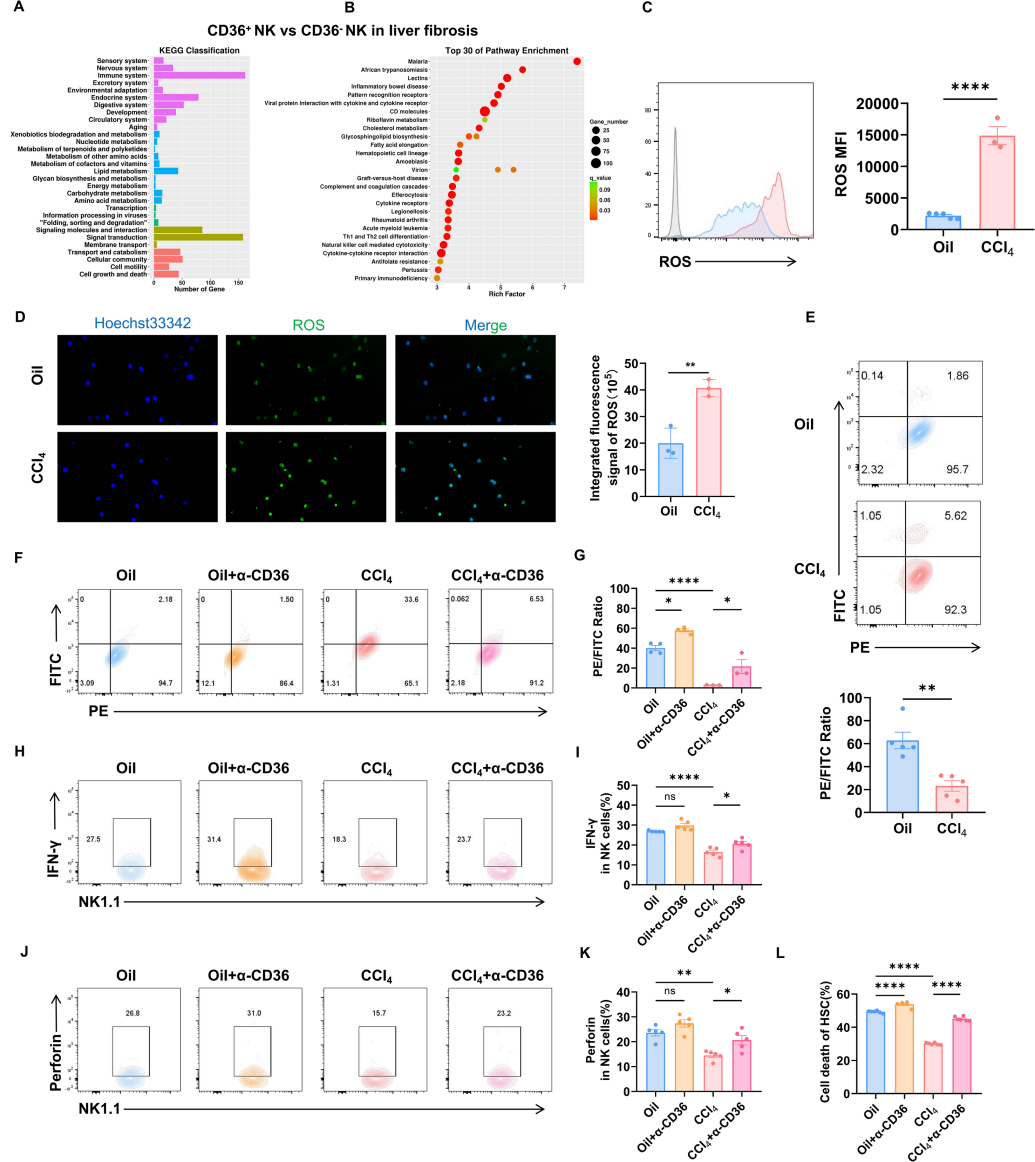
CD36 regulate lipid peroxidation in NK cells. **(A)** Top 30 most enriched KEGG classifications associated with DEGs (CD36^+^ NK vs CD36^-^ NK from fibrotic livers). **(B)** Top 30 most enriched KEGG pathways associated with DEGs (CD36^+^ NK vs CD36^-^ NK from fibrotic livers). **(C)** Liver NK cells from WT or fibrotic mice were analyzed for ROS after CCl_4_ treatment for 4 weeks. **(D)** Purified NK cells from WT or fibrotic mice were analyzed for ROS after CCl_4_ treatment for 4 weeks. **(E)** Liver NK cells from WT or fibrotic mice were analyzed for lipid peroxidation. A decreased PE/FITC ratio is indicative of elevated lipid peroxidation levels. **(F, G)** NK cells from the Oil or liver fibrosis group were incubated with or without 10 μg/mL αCD36 for 18 h. Lipid peroxidation was analyzed using flow cytometry. **(H-K)** Liver mononuclear cells (1×10^6^) were incubated with or without 10 μg/mL α-CD36 for 18 h and then analyzed for the expression of IFN-γ and perforin of NK cells. **(L)** HSCs (5×10^3^) were seeded for 24 h and then cultured with serum-free RPMI 1640 for 6 h to activate HSCs. NK cells (5×10^4^) from the Oil or liver fibrosis group were pretreated with 10 μg/mL α-CD36 for 6 h in complete 1640 medium. The treated NK cells were co-cultured with HSCs for another 12 h. The lactate dehydrogenase release assay was used to determine the cytotoxicity of NK cells against HSCs. Data are presented as means ± SEM. *, *p* < 0.05; **, *p* < 0.01; ****, *p* < 0.0001.

Ma et al. demonstrated that CD36 on CD8^+^ T cells promoted fatty acid uptake and induced lipid peroxidation (13). We found elevated cholesterol and non-esterified fatty acid (NEFA) in the serum and liver tissue after CCL_4_-treatment (**Supplementary Figures 4A–C**). However, the impact of CD36 on NK cell function remained unclear. To investigate this, we sorted CD36^+^ NK and CD36^-^ NK cells from the fibrotic livers for RNA sequencing. The KEGG classification revealed that CD36 significantly impacted the lipid metabolism class in NK cells (**Figure 3A**). In addition, there was significant enrichment of pathways that included cholesterol metabolism, glycosphingolipid biosynthesis, and fatty acid elongation (**Figure 3B**). Lipid metabolism influences ROS levels via substrate availability (e.g., FFAs and PUFAs), energy metabolism (e.g., mitochondrial function), antioxidant systems (e.g., NADPH and GSH), and signaling (e.g., inflammatory cytokines and enzyme activity) (14). We therefore measured the ROS change in NK cells. The flow cytometry and immunofluorescence showed significantly elevated ROS in liver NK cells from CCl_4_-treated mice (**Figures 3C, D**). This ROS accumulation can trigger lipid peroxidation, which was indeed significantly increased in liver NK cells from the CCl_4_-treated mice (**Figure 3E**). To determine if CD36 mediated NK cell lipid peroxidation, we co-cultured liver NK cells with a CD36 blocking antibody (α-CD36). This treatment effectively reduced lipid peroxidation in the NK cells (**Figures 3F, G**). To further explore the CD36 function, we co-cultured liver lymphocytes with α-CD36. This restored IFN-γ and perforin secretion by NK cells compared to those from the CCl_4_-treated mice alone (**Figures 3H–K**). Additionally, the CD36 blockage rescued the cytotoxic capacity of NK cells against HSCs (**Figure 3L**). These results indicated that CD36 mediated lipid peroxidation in hepatic NK cells, and CD36 blockage rescued the NK cell function.

### CD36 blockage improved ferroptosis and rescued the NK function

**Figure 4:**
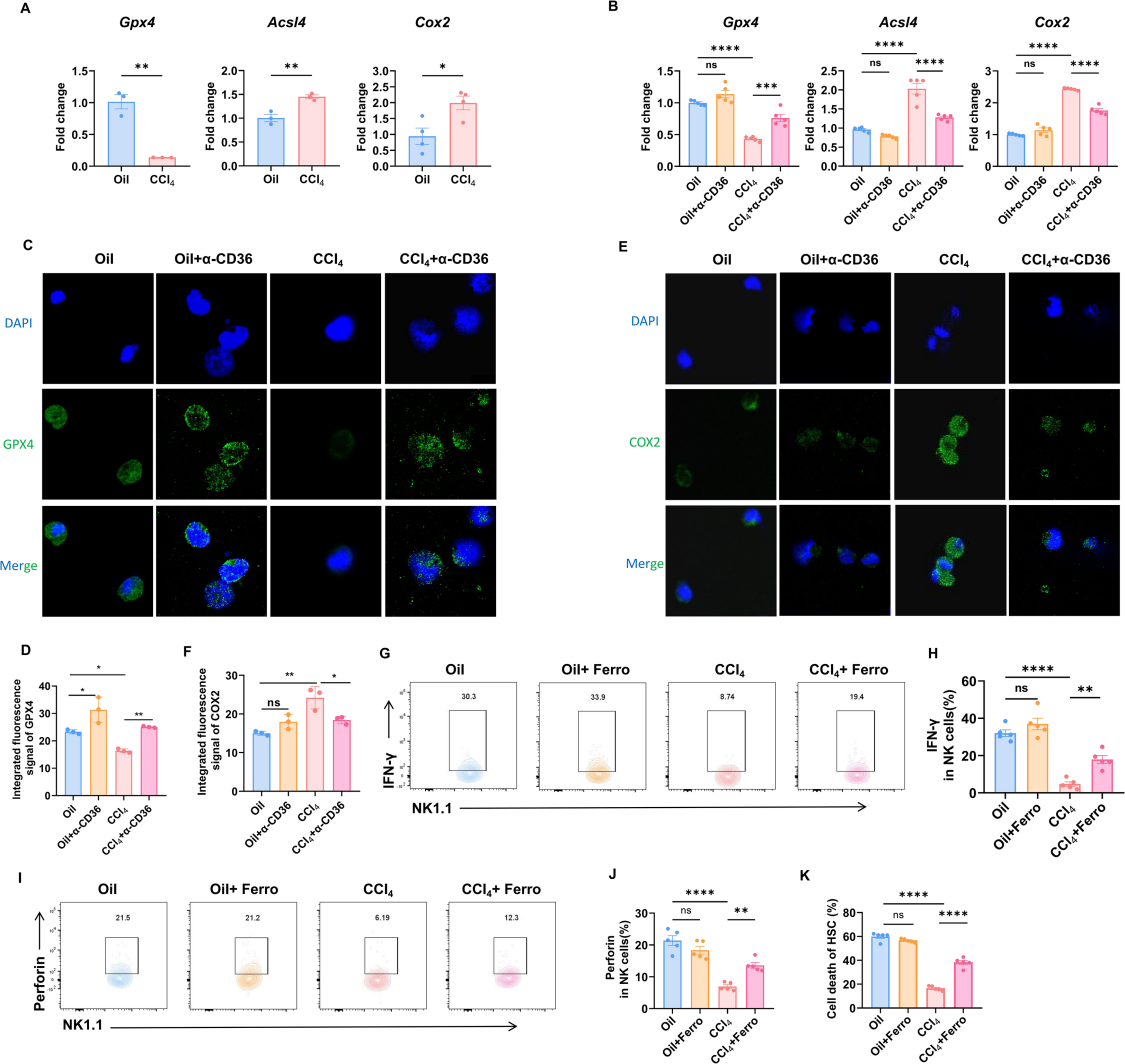
CD36 mediates ferroptosis and the ferroptosis inhibitor rescues cytotoxic cytokine production in NK cells of liver fibrotic mice. **(A)** mRNA expression of ferroptosis-related genes in primary NK cells from the livers of CCl_4_-treated mice compared with the control mice. **(B)** NK cells from the livers of CCl_4_-treated mice and control mice treated with or without α-CD36 were analyzed for ferroptosis-related genes. **(C-F)** Immunofluorescence images of GPX4 and COX2 in NK cells from CCl_4_-treated mice and control mice. Liver mononuclear cells (1×10^6^) were incubated with or without 10 μM Ferrostatin-1 for 18 h and then analyzed for the expression of IFN-γ **(G, H)** and perforin **(I, J)** of NK cells. **(K)** HSCs (5×10^3^) were seeded for 24 h and then cultured with serum-free RPMI 1640 for 6 h to activate HSCs. NK cells (5×10^4^) from the Oil or liver fibrosis group were pretreated with 10 μM ferrostatin-1 for 6 h in complete 1640 medium. The treated NK cells were co-cultured with HSCs for another 12 h. The lactate dehydrogenase release assay was used to determine the cytotoxicity of NK cells against HSCs. Data are presented as means ± SEM. *, *p* < 0.05; **, *p* < 0.01; ****, *p* < 0.0001.

Increased intracellular lipid peroxidation can induce ferroptosis, thereby affecting cellular function. We examined the expression of ferroptosis-related genes in NK cells during liver fibrosis. Compared to NK cells from the livers of mice in the control group, those from the livers of mice in the CCl_4_-induced group showed significantly elevated expression of the pro-ferroptosis genes *Acsl4* and *Cox2* and a significantly reduced expression of the ferroptosis-inhibitory gene *Gpx4* (**Figure 4A**). We hypothesized that NK cells might undergo ferroptosis via CD36-mediated lipid peroxidation during liver fibrosis. This would lead to their reduced activity and functional impairment. To test this, liver NK cells were isolated and cultured with α-CD36 for 18 h *in vitro*. This was followed by a ferroptosis-related gene expression analysis. The results demonstrated that, compared to NK cells from the CCl_4_-induced mice, those treated with the CD36 blocking antibody exhibited a significant decrease in *Acsl4* and *Cox2* expression and a significant increase in *Gpx4* expression (**Figure 4B**). This trend was observed in the immunofluorescence assays (**Figures 4C–F**). These results indicated that CD36 blockage effectively inhibited ferroptosis in hepatic NK cells during fibrosis. To further investigate the impact of ferroptosis on NK cell function, isolated lymphocytes were treated *in vitro* with a ferroptosis inhibitor for 18 h. Compared to the NK cells from the CCl_4_-induced mice, those treated with the ferroptosis inhibitor showed a significant recovery in their ability to secrete both IFN-γ and perforin (**Figures 4G–J**). This effectively restored their cytotoxicity against HSCs (**Figure 4K**). These results strongly suggested that CD36 impaired NK cell function by inducing ferroptosis.

### Liver fibrosis was improved by the adaptive transfer of CD36^-^ NK cells

**Figure 5:**
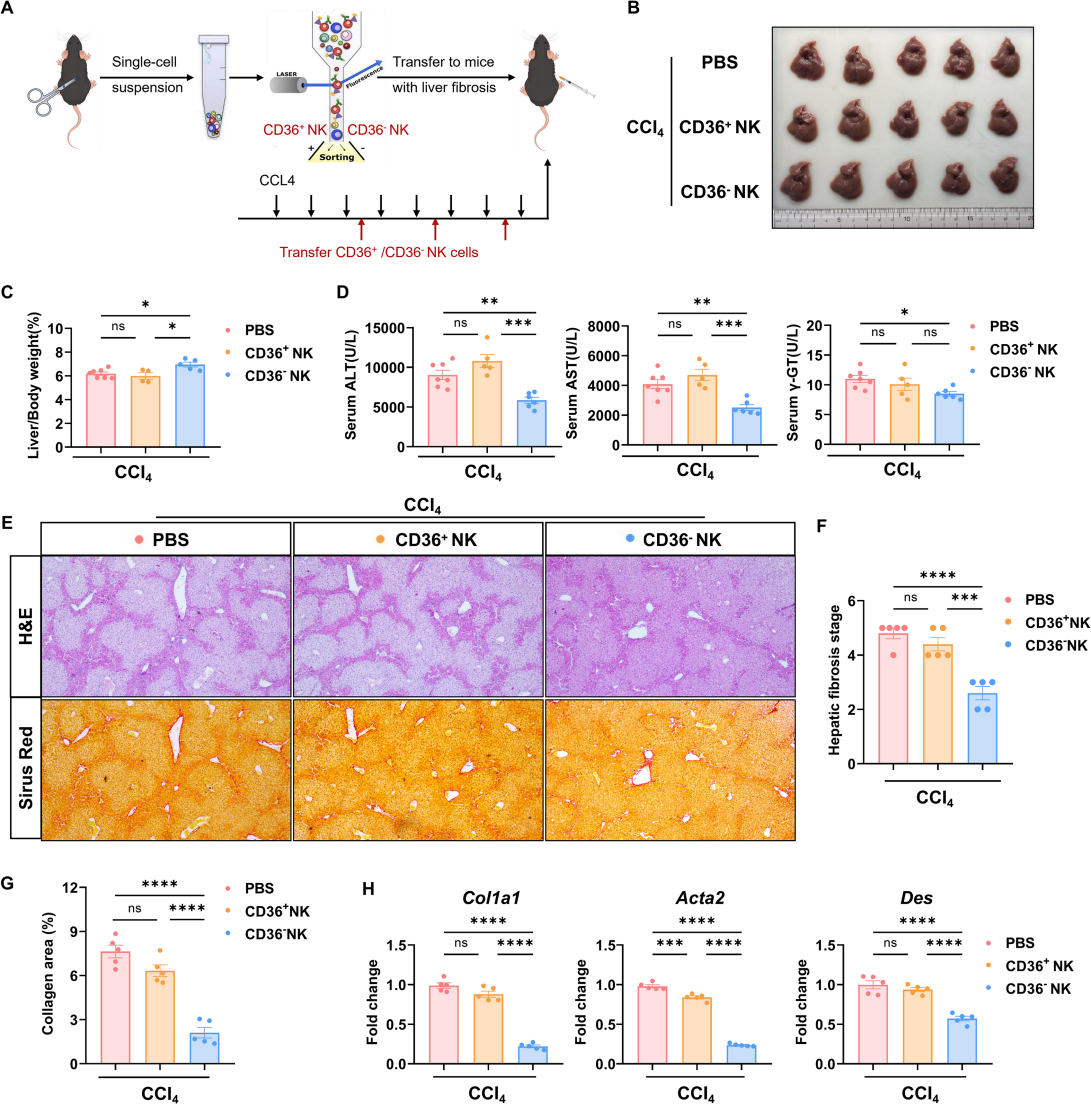
CD36^-^ NK cells alleviate CCl_4_-induced liver fibrosis in mice. **(A)** Schematic of CD36^+^/CD36^-^ NK cell transfer into CCl_4_-challenged mice. **(B)** Representative images of the fibrotic liver transferred by the indicated NK cells (1×10^5^) three times. **(C)** Liver/body weights of the fibrotic liver transferred by the indicated NK cells (1×10^5^). **(D)** Serum ALT, AST, and γ-GT of the indicated mice were analyzed. **(E-G)** H&E (100×) and Sirius red staining (100×) of the livers from the CCl_4_-challenged mice transferred with the indicated NK cells. **(H)** mRNA expression of fibrosis-related genes (*Col1a1*, *Acta2*, and *Des*) in liver tissues. Data are presented as means ± SEM. *, *p* < 0.05; **, *p* < 0.01; ***, *p* < 0.001; ****, *p* < 0.0001.

To verify whether the targeting of CD36 on NK cells would affect liver fibrosis progression, we transferred purified CD36^+^ NK cells and CD36^-^ NK cells via the tail vein into fibrotic mice. We then evaluated the capacity of CD36^-^ NK cells and CD36^+^ NK cells to alleviate liver fibrosis (**Figure 5A**). There were no significant differences in the hepatic pathological features due to the cell transfer of CD36^+^ NK cells or CD36^-^ NK cells (**Figure 5B**). To our surprise, the liver weight/body weight ratio was slightly increased in mice transferred with CD36^-^ NK cells (**Figure 5C**). Moreover, compared with mice transferred with CD36^+^ NK cells, the serum levels of alanine transaminase (ALT) and aspartate transaminase (AST) were remarkably decreased in those mice transferred with CD36^-^ NK cells (**Figure 5D**). Hematoxylin and eosin (H&E) staining demonstrated that the CD36^-^ NK cell notably improved liver fibrosis in mice (**Figures 5E, F**), while Sirius Red staining revealed less collagen deposition in the livers of mice transferred with CD36^-^ NK cells (**Figures 5E, G**). Additionally, we detected the expression of fibrosis-related genes (*Col1a1, Acta2*, and *Des*) in the liver tissues from each group. Compared with the control group, the expression of these fibrosis-related genes was significantly downregulated in the liver tissues of mice transferred with CD36^-^ NK cells, whereas no obvious change was observed in mice transferred with CD36^+^ NK cells (**Figure 5H**). These data showed that NK cells that did not express CD36 had a stronger capacity to improve liver fibrosis progression.

## Discussion

In this study, we revealed the mechanism that underlies NK cell dysfunction during liver fibrosis and explored potential approaches to reverse this dysfunction and enhance NK cell activity. Ultimately, these strategies were designed to strengthen their capacity to eliminate activated hepatic HSCs and improve their antifibrotic efficacy. We revealed that NK cell dysfunction during liver fibrosis was linked to CD36-mediated lipid peroxidation accumulation and subsequent ferroptosis. Notably, CD36 blockage effectively restored the NK cell activity during liver fibrosis progression. Furthermore, the adoptive transfer experiments revealed that CD36^-^ NK cells exerted superior *in vivo* antifibrotic activity, a trait absent in their CD36^+^ counterparts. These results suggested that the targeting of CD36 to modulate NK cell function is a viable strategy. The results also highlighted the critical role of CD36-mediated metabolic reprogramming in driving NK cell dysfunction during liver fibrosis. Thus, future therapeutic strategies should prioritize the development of selective interventions to restore NK cell activity, particularly their ferroptosis resistance and HSC-killing capacity. This would be preferable to the adoption of non-specific immune modulation approaches.

Hepatic fibrosis has been identified as an independent risk factor for the development of hepatocellular carcinoma (HCC). HSCs are key effector cells in hepatic fibrosis, and they play a pivotal role in HCC development. For a long time, they have served as a key research focus in exploring the mechanisms by which hepatic fibrosis fosters a pro-tumorigenic microenvironment within the liver. Upon activation, hepatic stellate cells (HSCs) migrate to regions of liver damage, where they release large amounts of inflammatory factors and constituents of the extracellular matrix (ECM) (15). The targeting of HSCs might be an effective method to improve liver fibrosis progression. The diminished cytotoxicity activity of NK cells against HSCs is a critical contributing factor during hepatic fibrosis progression to HCC (16, 17). How to rescue NK function has attracted our attention. Some immune cells have been reported to interact with NK cells and impact their function. Mucosal-associated invariant T (MAIT) cells can alleviate liver fibrosis through potentiating the cytotoxic function of natural killer (NK) cells (18). Similarly, γδT cells exert an anti-fibrotic effect by exerting direct cytolytic activity and augmenting NK cell-mediated cytotoxicity toward hepatic stellate cells (HSCs) (16). Moreover, M1-polarized bone marrow-derived macrophages (BMDMs) display more potent therapeutic efficacy by remodeling the hepatic microenvironment; this process promotes the recruitment and functional modulation of endogenous macrophages and NK cells, which in turn induce HSC apoptosis and inhibit the progression of hepatic fibrosis (19). Moreover, some molecules can be targeted to regulate the function and apoptosis of NK cells. Uncoupling protein 1 (UCP1) catalyzes the leakage of protons across the mitochondrial inner membrane for thermogenesis, the decrease of which promotes NK cell necroptosis (20). This includes non-alcoholic steatohepatitis (NASH) progression to fibrosis. Activation of metabotropic glutamate receptor 5 (mGluR5) within natural killer (NK) cells mitigates liver fibrosis in both murine models and human systems by elevating cytotoxic activity and boosting IFN-γ secretion (21). Additionally, EP3 is indispensable for the adhesion and cytotoxic capacity of NK cells against hepatic stellate cells (HSCs). EP3 deficiency compromises the adhesive and cytotoxic functions of DP NK cells toward HSCs by interfering with the interaction between integrin subunit alpha 4 (ITGA4) and vascular cell adhesion molecule-1 (VCAM1) (22).These studies indicate that the targeting of NK cells may be an effective method to attenuate liver fibrosis progression, but the target molecules have not been fully explored. In our study, we found that CD36 upregulation inhibited NK function with decreased cytotoxicity against HSCs and cytokine production. CD36 blockage reversed this process, suggesting that CD36 could serve as a new target to improve NK cytotoxicity toward HSCs.

CD36 is a membrane glycoprotein that has been extensively studied in various mammalian cells, such as adipocytes, macrophages, and hepatocytes (23, 24). In recent years, an increasing amount of research has focused on CD36-mediated dysfunctions in immune cells. Previous studies have documented that CD36 is capable of inducing ferroptosis in tumor-infiltrating CD8^+^ T lymphocytes. As reported by Ma X et al., CD8^+^ T cells internalize fatty acids through CD36, which in turn elicits lipid peroxidation in these T cells. This cascade of events ultimately promotes ferroptosis and diminishes the secretion of cytotoxic cytokines, resulting in the loss of antitumor effector functions within the tumor microenvironment (TME). Notably, blocking CD36-mediated ferroptosis, particularly when combined with immune checkpoint blockade therapy, markedly potentiates the antitumor efficacy of CD8^+^ T cells (13). Interestingly, CD36 has been recently reported to have another function where it promoted the antigen-presenting phenotype of myeloid-derived suppressor cells (MDSCs) by facilitating the uptake and integration of B-cell membrane components (25). However, how CD36 mediates NK cell dysfunction remains not fully explored. In our study, we demonstrated that CD36-mediated accumulation of lipid peroxidation ultimately led to ferroptosis that impaired the cytotoxic function of NK cells during liver fibrosis and compromised their anti-fibrotic capacity. The transfer of CD36^-^ NK could improve liver fibrosis. Additionally, CD36 is also expressed in HSCs (26). CD36 signaling plays a significant pro-fibrotic role in the activation of HSCs to stimulate matrix synthesis (27). In summary, these data indicated that CD36 might be an effective target for liver fibrosis therapy.

NK cells are reported to divide into two subsets, conventional NK (cNK) and liver-resident NK (termed LrNK or liver ILC1) (11). They both can produce IFN-γ to prevent fibrosis in chronic liver diseases (28). The depletion of NK cells and ILC1 lead to MASLD progression by promoting the polarization of macrophages (29). However, how these two subsets are regulated by CD36 during liver fibrosis remains undocumented. In our study, we found that CD36 expression was upregulated in both cNK and LrNK cells, with a significantly greater increase observed in LrNK cells. Interestingly, we observed that CD36^-^ NK cells expressed higher levels of *Eomes*, *Ncr1*, *Tbx21*, *Ccl5*, *Itga2*, and *Il18r1*—signature genes associated with cNK cells (data not shown). Thus, we speculated that cNK cells may, to some extent, be able to resist the upregulation of CD36 in liver fibrosis.

Ferroptosis is an iron-dependent form of cell death driven by lipid peroxidation, which may play a role in the pathology of certain degenerative diseases (30). While the role of iron metabolism in ferroptosis regulation has been firmly established, a growing body of research has indicated that numerous other metabolic networks are also closely intertwined with the regulation of this regulated cell death modality. Key examples of such pathways include the GSH–GPX4 axis, GCH1-BH4-DHFR axis, and FSP1–CoQ axis (31). Although some studies have reported the potential effect of targeting ferroptosis of immune cells in liver disease (32–34), there were only few studies that have reported how ferroptosis influences the NK function. Sophie M Poznanski et al. reported that NK cells exhibit a glucose metabolism disorder within the TME that is accompanied by lipid peroxidation and oxidative damage. Nrf2 pathway activation can enhance the antioxidant capacity of NK cells, thereby improving their anti-tumor efficacy (35). Our study focused on the occurrence of ferroptosis in hepatic NK cells during liver fibrosis and its impact on their anti-fibrotic capacity. We found that CD36 might have influenced the lipid metabolism of NK cells, which led to ferroptosis of NK cells in liver fibrosis. Blocking CD36 *in vitro* can reduce ferroptosis levels and restore the anti-fibrotic capacity of NK cells during liver fibrosis. Blocking ferroptosis can restore the cytotoxicity of NK cells against HSCs. This led us to investigate whether similar effects occur *in vivo*. We observed that CD36^-^ NK cells exhibited stronger anti-fibrotic ability *in vivo*, a capability that was not present in CD36^+^ NK cells. Currently, clinical management of liver fibrosis primarily focuses on eliminating causative factors and symptomatic treatment, with no specific therapeutic drugs available. This study reveals that CD36^-^ NK cells possess unique anti-fibrotic properties in liver fibrotic mice and that they can reverse the progression of liver fibrosis. Although the findings provide a new target to improve NK function in liver fibrosis, we lack direct experimental evidence regarding the role of NK cell-specific CD36 deletion in the progression of liver fibrosis, which needs to be further investigated.

In summary, we revealed that CD36 plays a critical role in regulating NK cells. Our data demonstrated that CD36 mediated NK cell ferroptosis. Blockade of CD36 in NK cells could be a therapeutic strategy for combating liver fibrosis. These findings may provide new insights into cell immunotherapy for liver fibrosis.

## Data Availability

The original contributions presented in the study are publicly available. This data can be found here: NCBI GEO, accession GSE330049.
