# Supplementary material for: CD36 aggravates ferroptosis in NK cells and dampens their anti-fibrotic activity in the liver
Source: Front Immunol. 2026 May 14;17:1825015. doi: 10.3389/fimmu.2026.1825015 (PMC13216501; doi:10.3389/fimmu.2026.1825015)
Supplement: Supplementary file 1 [file DataSheet1.docx]

**Supporting information**


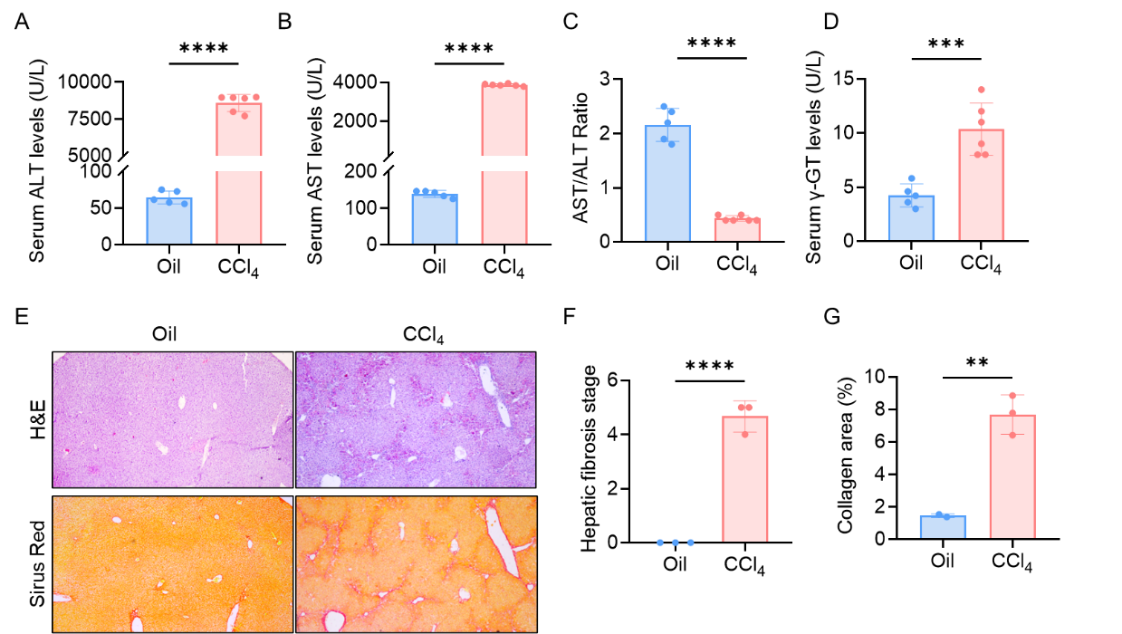


**Fig. S1. Establishment of a mouse model of CCl₄‑induced hepatic fibrosis.**

(A-D) Serum levels of ALT, AST, AST/ALT and γ-GT in the control group and mice treated with CCl_4_. (E) Representative images of H&E and Sirius Red staining in the control group and mice treated with CCl_4_. (F-G) Statistical analysis of fibrosis degree is shown on the right. H&E hematoxylin and eosin, ALT alanine aminotransferase, AST aspartate aminotransferase, CCl_4_ carbon tetrachloride.

**
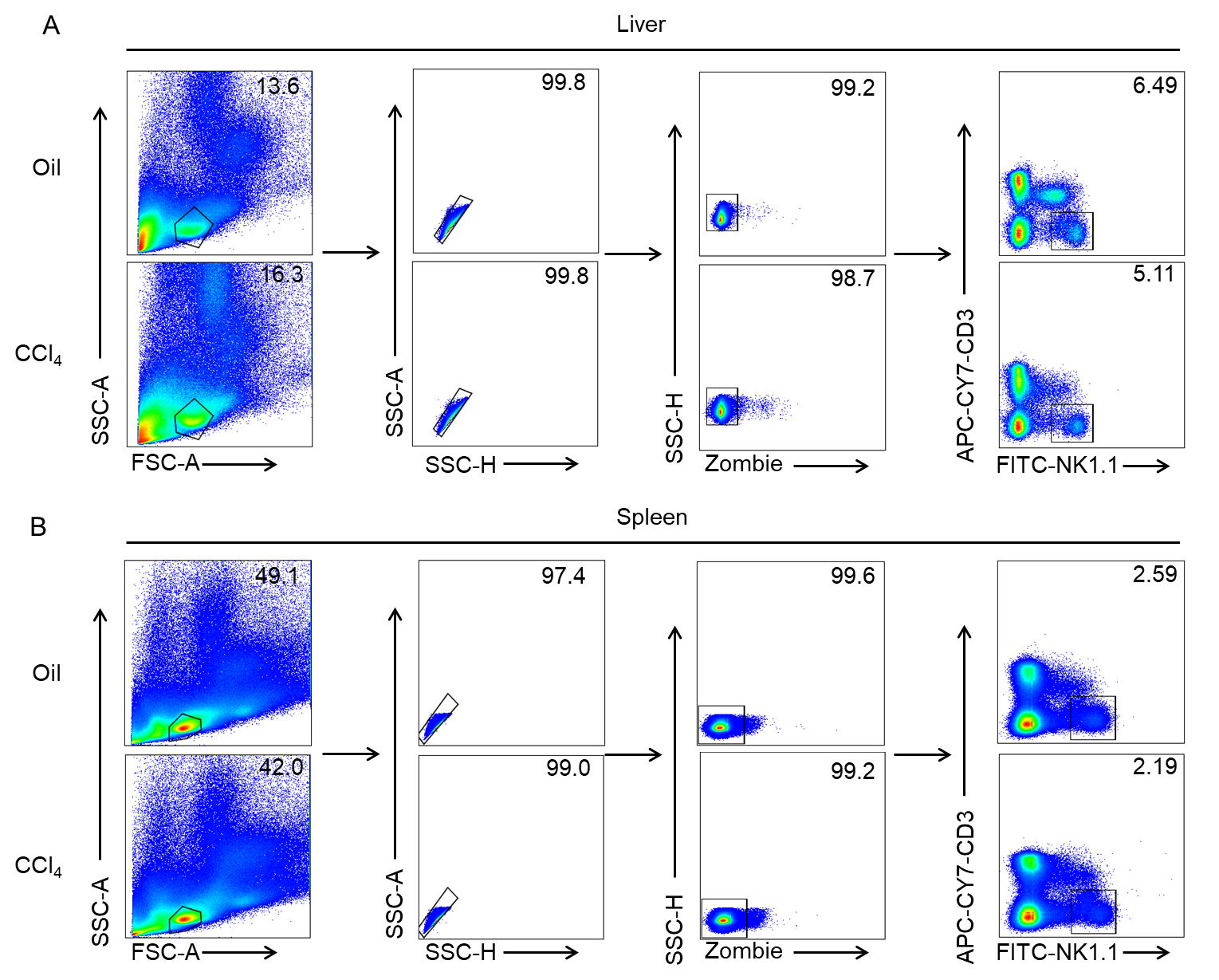
**

**Fig. S2. The flow cytometry gating strategy for hepatic or splenic NK cells.**

Lymphocytes of liver (A) and spleen (B) were identified by forward and side scatter (FSC-A vs. SSC-A), and single cells were identified (SSC-A vs. SSC-H). Next, dead cells (Zombie^+^) were excluded, and NK cells were defined as CD3^-^ NK1.1^+^.


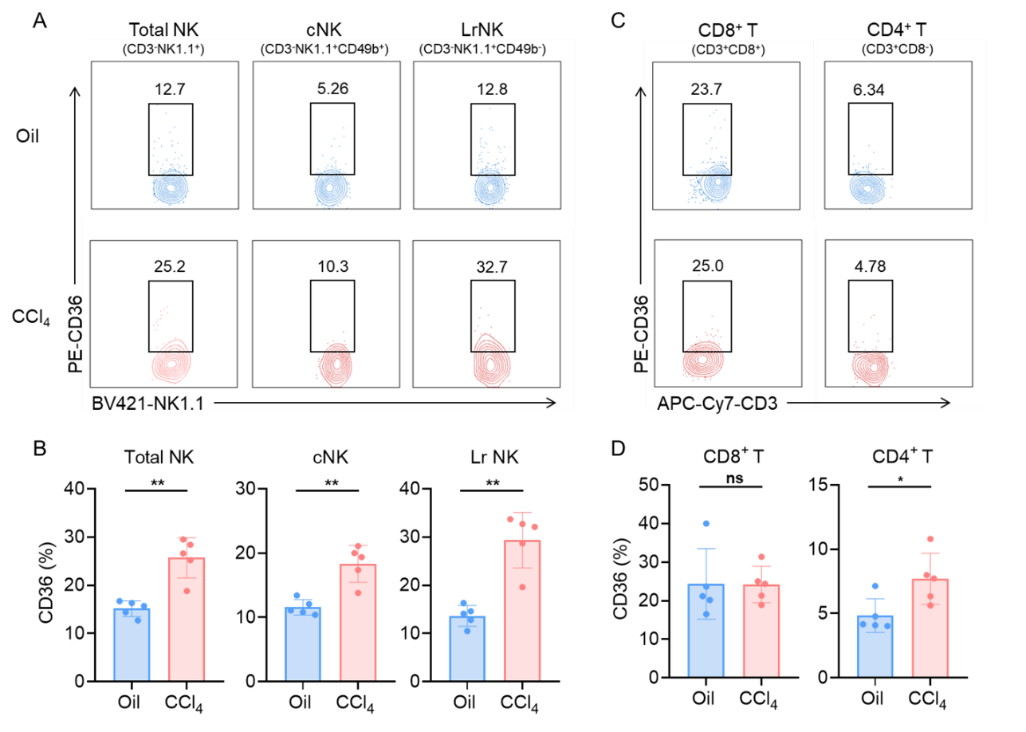


**Fig. S3. CD36 expression on hepatic MNCs after** **CCl₄‑induced hepatic fibrosis.**

WT mice was treated with CCl_4_ for two weeks. (A, B) Expression of CD36 on NK subsets. (C, D) Expression of CD36 on T cell subsets.


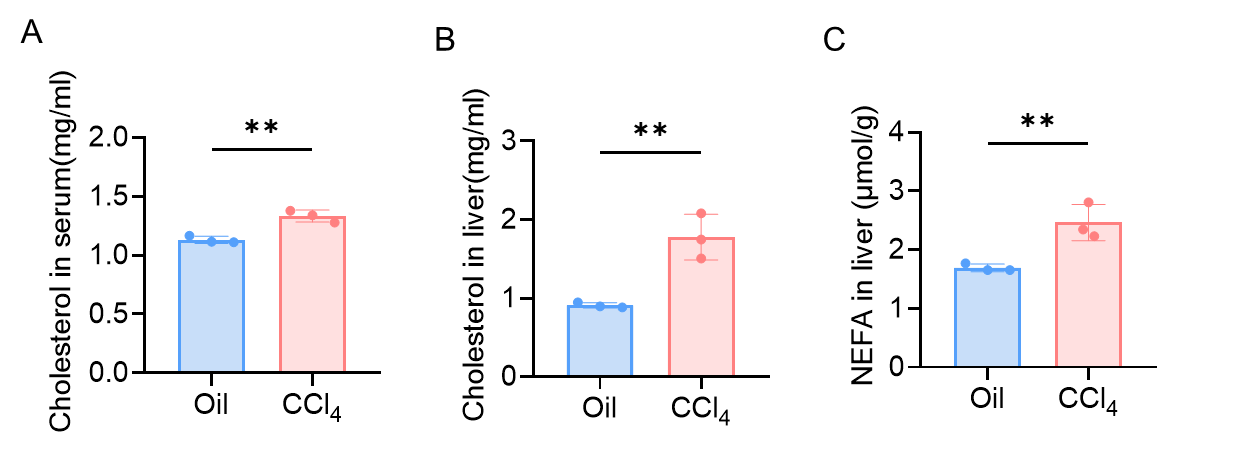


**Fig. S4. Elevated cholesterol and non‑esterified fatty acids in hepatic fibrosis mice** (A) Serum levels of cholesterol in the control group and mice treated with CCl_4_. (B) Hepatic cholesterol in the control group and mice treated with CCl_4_. (C) Hepatic NEFA in the control group and mice treated with CCl_4_.
